# Is subcutaneous adipose tissue expansion in people living with lipedema healthier and reflected by circulating parameters?

**DOI:** 10.3389/fendo.2022.1000094

**Published:** 2022-10-31

**Authors:** Pamela A. Nono Nankam, Manuel Cornely, Nora Klöting, Matthias Blüher

**Affiliations:** ^1^ Helmholtz Institute for Metabolic, Obesity and Vascular Research (HI-MAG), Helmholtz Zentrum München, University Hospital Leipzig, University of Leipzig, Leipzig, Germany; ^2^ Basic Scientific Research of Lymphological Diseases and Patient-oriented Improvement of Diagnosis and Treatment Ly.Search GmbH, Cologne, Germany; ^3^ Medical Department III – Endocrinology, Nephrology, Rheumatology, University of Leipzig Medical Center, Leipzig, Germany

**Keywords:** lipedema, adipose tissue, inflammation, oxidative stress, glucose metabolism

## Abstract

Lipedema may be considered a model for healthy expandability of subcutaneous adipose tissue (SAT). This condition is characterized by the disproportional and symmetrical SAT accumulation in the lower-body parts and extremities, avoiding the abdominal area. There are no circulating biomarkers facilitating the diagnosis of lipedema. We tested the hypothesis that women living with lipedema present a distinct pattern of circulating parameters compared to age- and BMI-matched women. In 26 women (Age 48.3 ± 13.9 years, BMI 32.6 ± 5.8 kg/m2; lipedema group: n=13; control group: n=13), we assessed circulating parameters of glucose and lipid metabolism, inflammation, oxidative stress, sex hormones and a proteomics panel. We find that women with lipedema have better glucose metabolism regulation represented by lower HbA1c (5.55 ± 0.62%) compared to controls (6.73 ± 0.85%; p<0.001); and higher adiponectin levels (lipedema: 4.69 ± 1.99 mmol/l; control: 3.28 ± 1.00 mmol/l; p=0.038). Despite normal glycemic parameters, women with lipedema have significantly higher levels of total cholesterol (5.84 ± 0.70 mmol/L vs 4.55 ± 0.77 mmol/L in control; p<0.001), LDL-C (3.38 ± 0.68 mmol/L vs 2.38 ± 0.66 mmol/L in control; p=0.002), as well as higher circulating inflammation (top 6 based on p-values: TNFSF14, CASP8, EN-RAGE, EIF4EBP1, ADA, MCP-1) and oxidative stress markers (malondialdehyde, superoxide dismutase and catalase). Our findings suggest that the expected association between activation of inflammatory and oxidative stress pathways and impaired glucose metabolism are counterbalanced by protective factors in lipedema.

## Introduction

Lipedema is a loose connective tissue disease characterized by the disproportional and excessive accumulation of fibrotic subcutaneous adipose tissue (SAT) mainly around the buttocks, hips and limbs (1–4). Therefore, lipedema could serve as a model to investigate mechanisms linking impaired SAT expandability to increased cardio-metabolic risk. In this context, it has been shown that higher leg fat accumulation and low trunk fat are associated with lower cardiovascular disease risk in postmenopausal women with normal BMI (5). Consistently, lipedema has been associated with a lower risk for diabetes and other cardio-metabolic dysfunctions (4, 6). Indeed, based on clinical observations, the excessive SAT accumulation in lipedema is predominant in the arms and lower-body parts (legs), sparing the abdominal area contrarily to obesity (7–9).

Lipedema is mostly diagnosed in women and its onset and development are generally recorded in periods of hormonal, weight and shape shifts such as during puberty, pregnancy, childbirth and menopause (1). The main clinical component concomitant to the increase of SAT mass, but distinguishing lipedema from obesity is the reported pain (“*painful fat*”), which together with the increased tissue fibrosis is speculated to be a consequence of high inflammation in lipedema tissue (1, 10, 11). Moreover, the reduction of AT mass by various weight loss measures is less effective in lipedema patients compared to obesity, which severely affects patients’ quality of life and leads to psychological problems and an increased risk of suicide (3, 4, 8, 10, 12, 13). Although lipedema was described decades ago (14), the relative scarcity of data and understanding of the mechanisms associated with its onset, progression and clinical manifestation is surprising. Furthermore, there is currently no epidemiology-based data on the prevalence of lipedema, which is estimated to vary between 7 – 18% of women across different studies’ cohorts (4, 8, 15–18).

Lipedema is one of the most often under/misdiagnosed fat accumulation disorders (9, 19). This is mainly due to the similarities in phenotypic features with other excessive fat accumulation like obesity (13). However, the dysregulations related to excessive SAT accumulation have been well described in the context of obesity (20). Indeed, adipose tissue (AT) dysfunction may develop in response to progressive and continuous lipids storage in obesity (20). This is characterized among others by impaired adipogenesis (21, 22) and angiogenesis (23, 24), fibrosis (20, 25) and hypoxia (26, 27), dysregulated adipokine secretion (27–29), AT inflammation (26, 28, 30) as well as oxidative stress (27, 31). These metabolic processes can interfere with insulin signalling pathways, contributing to the development of insulin resistance, type 2 diabetes (T2D) and other obesity-associated metabolic diseases (20, 32–34). Some of these dysregulations have also been described in lipedema, including dysregulated adipogenesis (35, 36) and angiogenesis (9, 37), fibrosis and hypoxia (38, 39), immune cell infiltration and AT inflammation (35, 37, 40, 41). However, it remains unknown whether and to which extent the excessive expansion of AT in lipedema influences whole-body metabolic health in these patients.

With regard to the similarities in some components of AT dysfunction described in both conditions, the resulting obesity-associated metabolic dysregulations could be expected in lipedema as well. Indeed, adipocyte hypertrophy in obesity is associated with increased systemic insulin resistance, and inflammatory and oxidative stress (33, 34, 42). However, lipedema patients are reported with a relatively lower incidence of T2D although at advanced stages these patients seem to present a higher risk for developing obesity and cardiovascular diseases (8, 17). This suggests dissimilar mechanisms associating SAT accumulation to metabolic health status between lipedema and obesity. AT exerts its effect on whole-body homeostasis partially through the endocrine action of adipocytokines and lipids released into the circulation, which thereby affects the function of other tissues (43). For instance, increased AT macrophage infiltration in obesity influences local and systemic inflammation through dysregulated cytokine secretion (29, 44). Adipose-derived biomolecules may therefore represent a valuable target for an early diagnosis and treatment of lipedema. We hypothesize that circulating factors may mediate the beneficial effects of adipose tissue expandability to protect against cardio-metabolic diseases. Therefore, we aimed to characterize circulating biomarkers of glucose and lipid metabolism, inflammation and oxidative stress in lipedema patients, and evaluate the association between these markers and metabolic status. The outcomes of this study would allow profiling of systemic biomarkers dysregulated in lipedema and improve the current understanding of the mechanisms underlying the clinical manifestation of this condition.

## Methods

### Study participants

To investigate the molecular alterations in lipedema, 13 women diagnosed with lipedema aged from 24 to 71 years, with a body mass index (BMI) from 23.7 to 41.5 kg/m^2^ were enrolled in this study. These women were clinically diagnosed by two independent specialists in lymphology and related conditions based on defined clinical criteria (1, 16, 45). As there is currently no consensus to clearly categorize lipedema patients, the diagnosis relies on the reported symptoms and clinical observations, with no absolute phenotypic features or confirmatory tests. The control group was selected from women treated in our obesity outpatients clinic (n=13) to be age- and BMI-matched to the lipedema patients, but without any clinical symptoms of lipedema. The study was performed in agreement with the Declaration of Helsinki and approved by the Ethics Committee of the University of Leipzig (approval number: 159-12-21052012 and 017/12-ek). All individuals gave written informed consent before participating in the study.

### Basic anthropometry and sample collection

Basic anthropometric measures (weight and height) were recorded from all participants. Limb volumes were measured in lipedema patients using a perometer (Pero-System Meters Ltd. Germany) in lipedema patients. To measure circulating biomarkers, blood samples from participants of both groups were collected between 08 - 10 am in EDTA tubes after overnight fasting. EDTA-blood samples were centrifuged for 10 minutes at room temperature, at 3260g (sigma 2-16P, Germany), and plasma samples as well as red blood cells sub-fraction were aliquot and stored at -20°C.

### Plasma parameter analyses

Biomarkers of glucose and lipid metabolism, liver function, inflammation and oxidative stress as well as sex hormones were assessed in plasma samples of patients from both groups using commercially available kits according to the manufacturer’s instructions. Briefly, plasma concentrations of glucose (ACN8717), triglycerides (ACN7881), total cholesterol (ACN8798), low-density lipoprotein cholesterol (LDL-C; ACN8552), high-density lipoprotein cholesterol (HDL-C; ACN8454), alanine transaminase (ALAT), aspartate aminotransferase (ASAT) and gamma-glutamyltransferase (gGT; ACN8220), as well as lipase (ACN789) were determined by homogeneous enzymatic colorimetric assays (COBAS 8000-c502 and c701; Roche Diagnostics, Germany). Chronic hyperglycemia was estimated by the measure of haemoglobin A1c (HbA1c) levels in full-blood samples using immunoturbidimetric assay (HITADO Super-ID-System; Germany). Insulin (10-1113-01; Mercodia, Uppsala, Sweden), C-peptide (10-1136-01; Mercodia), leptin (E07; Mediagnost, Reutlingen, Germany), adiponectin (AG-45A-0001YEK-KI01; AdipoGen Life Sciences, San Diego, CA, USA), tumor necrosis factor-alpha (TNF-α; HSTA00E; R&D Systems, Minnesota, Minneapolis, USA), as well as circulating concentrations of 17-β estradiol (ab108667; Abcam, Cambridge, UK) and progesterone (ab108670; Abcam) were measured using commercially available ELISA kits. Testosterone levels were measured by a liquid chromatography-tandem mass spectrometry (LC-MS/MS) method as previously described (46). The plasma concentration of C-reactive protein IV (CRP4) was assessed by particle-enhanced immunoturbidimetric assay (ACN8256; COBAS 8000-c701; Roche Diagnostics, Germany). To evaluate the oxidative stress status, plasma concentrations of malondialdehyde (MDA) (ab118970; Abcam), and activities of catalase (EIACATC; Invitrogen, Waltham, MA, USA) and superoxide dismutase (SOD) (EIASODC; Invitrogen) were assessed by colorimetric assays using commercially available kits.

In an unbiased proteomics approach, 92 additional circulating parameters of inflammation were assessed using the Olink multiplex proximity extension assay (Olink^®^ Proteomics, Uppsala, Sweden). Briefly, 92 oligonucleotides labelled antibody probe pairs were incubated with one microliter of plasma. This results in labelled antibody pairs binding to their respective target proteins when present in the evaluated samples, and the formation of a PCR reporter sequence by a proximity-dependent DNA polymerization. The reporter sequence was subsequently amplified and quantified by real-time PCR (Fluidigm^®^ Biomark™ HD system, San Francisco, CA, USA). Potential intra-assay variability was controlled by analyzing all samples in a single homogeneous 96-well format.

### Statistical analyses

Data are reported as mean ± standard deviation (when normally distributed) or median – interquartile range (when skewed) for patients’ basic characteristics and systemic biomarkers concentrations. Skewed data (i.e. *p* values < 0.05 from Shapiro-Wilks normality test) were log-transformed before analyses. The comparison of the limb volumes of lipedema patients was performed using a paired *t-*test. The difference in anthropometry and systemic biochemical markers was analyzed using a Mann-Withney Wilcoxon Test and significance levels were set at *p* < 0.05. The associations between the significantly different biomarkers and anthropometry and clinical measures were evaluated using Pearson correlations.

Multiplex inflammatory protein levels were reported as normalized protein expression (NPX), an arbitrary unit on a log2 scale. The comparison of protein expression levels between the groups was performed using Mann-Whitney Wilcoxon Test, and *p* values were adjusted for multiple comparison testing using Benjamini-Hochberg (false discovery rate – FDR) method. The heatmap was generated *via* hierarchical clustering using pheatmap. The reported significance levels correspond to adjusted *p* values (*p_adj*) < 0.05. Gene ontology (GO) enrichment analysis was performed and enriched biological processes were considered significant for *p_adj <*0.01. The analyses were performed using R software (Version 4.1.1, R Foundation for Statistical Computing 2022).

## Results

### Cohort characteristics

Twenty-six age- and BMI-matched women were included in this study and categorized into a lipedema and control group (**Table 1**). Women with lipedema have been further classified into different stages. Nine out of 13 lipedema patients were at stage 3, two patients were at stage 2 and two patients were at stage 1 (**Table 1**). Interestingly, there was a trend for increasing age (**Table 1**) and BMI (*Stage 1: 24.36 ± 0.97 kg/m^2^, Stage 2: 26.81 ± 1.04 kg/m^2;^ Stage 3: 35.77 ± 3.61 kg/m^2^*) with increasing lipedema stages. The average volume of the arms was 3871 ml (± 517 ml) and that of the legs was 12061 ml (± 2021 ml) in the lipedema group. There were no significant differences between right and left limbs (arms: p=0.828 and legs: p=0.372; **Table 1**), supporting bilateral and symmetrical SAT accumulation as described in lipedema patients (1–4, 7, 8). There was no significant difference in age (p=0.980) and BMI (p=0.898) between participants from lipedema and control groups (**Table 1**).

**Table 1:** Patient’s phenotypes.

Characteristics	Control group (n = 13)	Lipedema group (n = 13)
Age (years)	48.26 ± 13.59	48.30 ± 14.70
BMI (kg/m^2^)	32.51 ± 6.01	32.64 ± 5.77
**Lipedema Stage**		
Stage 1 (n); Age (years)	–	2; 29.00 ± 1.41
Stage 2 (n); Age (years)	–	2; 46.50 ± 10.61
Stage 3 (n); Age (years)	–	9; 53.00 ± 13.82
**Limb’s volume**		
Perometry left leg (mL)	–	11991 ± 1950
Perometry right leg (mL)	–	12130 ± 1975
Perometry left arm (mL)	–	3877 ± 509
Perometry right arm (mL)	–	3866 ± 497

Data presented as mean ± SD. BMI, body mass index.

### Parameters of glucose metabolism and sex hormones

A panel of biologically plausible biomarkers in both lipedema and control groups are summarized in **Table 2**. Interestingly, we found lower HbA1c levels in patients with lipedema compared to controls (p<0.001). In addition, fasting insulin and adiponectin concentrations were higher in lipedema compared to the control group (p<0.05). We did not find significant differences in circulating leptin levels between the groups, reflecting the group matching for BMI and suggesting that differences in body fat mass were not significant between the groups (**Table 2**). Sample haemolysis interfered in the measurement of glucose concentrations which were therefore not reported and insulin sensitivity indices could not be calculated. None of the other measurements was affected by haemolysis, except for ASAT concentrations as reported below. There were no significant differences in circulating levels of C-peptide as well as sex hormones concentrations (17β-Estradiol, progesterone and testosterone) between the groups (p>0.05). Noteworthy, sex hormone concentrations were highly variable between the individuals in the same groups, potentially due to the difference in the menstruation cycle of patients, which was not considered at the time of sample collection, representing a limitation of this study. Despite this inter-individual variability in circulating sex hormone concentrations, there was a trend for higher levels of circulating progesterone (median approx. 3-fold higher) in lipedema compared to the control group (p=0.180; **Table 2**).

**Table 2:** Systemic metabolic parameters and biochemical markers.

Variables	Control group (n = 13)	Lipedema group (n = 13)	P Value
***Carbohydrate metabolism***			
Insulin (pmol/L)	25.51 ± 15.21	47.01 ± 21.37	0.014
C-Peptide (pmol/L)	585.64 ± 181.04	584.23 ± 215.68	0.626
HbA1c (%)	6.73 ± 0.85	5.55 ± 0.62	<0.001
***Sex hormones***			
Testosterone	0.93 (0.78 – 1.26)	0.83 (0.67 – 1.12)	0.424
17β-Estradiol (pmol/l)	88.42 (32.72 – 315.41)	74.39 (38.51 – 209.81)	0.712
Progesterone (nmol/l)	0.67 (0.41 – 1.37)	2.12 (0.74 – 2.51)	0.180
***Liver/Pancreas enzymes***			
ALAT (µkat/L)	0.19 ± 0.07	0.48 ± 0.13	<0.001
ASAT (µkat/L)	0.40 ± 0.10	0.58 ± 0.75	0.005*
gGT (µkat/L)	0.17 (0.13 – 0.30)	0.24 (0.18 – 0.38)	0.136
Lipase (µkat/L)	0.55 ± 0.19	0.56 ± 0.16	0.719
***Lipid profile***			
Total-cholesterol (mmol/L)	4.55 ± 0.77	5.84 ± 0.70	<0.001
HDL-C (mmol/L)	1.38 ± 0.28	1.52 ± 0.45	0.521
LDL-C (mmol/L)	2.38 ± 0.66	3.38 ± 0.68	0.002
Triglycerides (mmol/L)	1.24 ± 0.65	1.51 ± 0.70	0.369
***Adipokines***			
Adiponectin (µg/mL)	3.28 ± 1.00	4.69 ± 1.99	0.038
Leptin (ng/mL)	22.34 ± 16.17	26.15 ± 11.57	0.305
***Inflammation***			
TNFα (pg/mL)	1.55 ± 0.46	2.19 ± 0.71	0.020
CRP (mg/L)	2.09 (0.98 – 5.45)	3.25 (1.36 – 4.42)	0.290
***Oxidative stress***			
SOD activity (U/ml)	0.94 (0.86 – 0.99)	1.42 (1.22 – 1.53)	<0.001
Catalase activity (U/ml)	7.61 (5.57 – 16.28)	62.47 (57.57 – 63.71)	<0.0001
MDA concentration (nmol/ml)	0.49 ± 0.05	0.53 ± 0.05	0.012

Values are presented as means ± SD for normally distributed variables and median (interquartile range) for non-normally distributed variables; P < 0.05 represents significant differences between the groups from Mann-Whitney Wilcoxon Test. *p > 0.05 when adjusting for sample haemolysis index. HbA1c, Hemoglobin A1c; ALAT, Alanine-Aminotransferase; ASAT, Aspartate-Aminotransferase; gGT, Gamma-glutamyl transferase; HDL-C, High-density lipoprotein cholesterol; LDL-C, Low-density lipoprotein cholesterol; TNFα, Tumour Necrosis Factor-alpha; CRP, C-reactive protein; MDA, Malondialhedhyde; SOD, Superoxide dismutase.

### Lipid profile and liver enzymes

Lipid profile analyses revealed higher concentrations of total cholesterol and LDL-C in lipedema compared to control patients (p<0.01), without differences in triglycerides and HDL-C levels between the groups (**Table 2**). In addition, we assessed the hepatic function by measuring circulating levels of liver enzymes and found higher concentrations of ALAT and ASAT in lipedema compared to the control group (p<0.01) while gGT and lipase levels did not differ between the groups (**Table 2**). To verify that these changes might be lipedema-related rather than associated with the sex-hormone state of the study participants, we adjusted the significant differences in systemic metabolic circulating cholesterol and liver enzyme concentrations for patients’ age. The reported differences between the groups persisted after adjustment (ALAT: p<0.001; ASAT: p<0.01; total cholesterol: p<0.001; LDL-C: p=0.001). After adjusting these parameters for samples haemolysis index, ASAT concentration was no longer significantly different between the groups (p>0.05).

### Distinct levels of systemic oxidative stress markers in lipedema patients

Oxidative stress can be evaluated by the assessment of lipids peroxidation through surrogate measures of biomolecules such as MDA concentrations in biological materials (31). Catalase and SOD represent the first lines of oxidative defence as antioxidant enzymes and can also be measured to evaluate oxidative stress (31). We found significantly higher MDA concentration and catalase and SOD activities in lipedema compared to the control group (p<0.01; **Table 2**). The cytokine TNFα is one of the most studied markers of inflammation and has been associated with increased oxidative stress (47). Concomitant with higher oxidative stress parameters, we found higher TNFα concentrations in lipedema compared to the control group (p<0.05; **Table 2**). In contrast, we did not find differences in circulating CRP between the groups.

### Correlations between systemic biochemical markers and metabolic parameters

**Figure 1:**
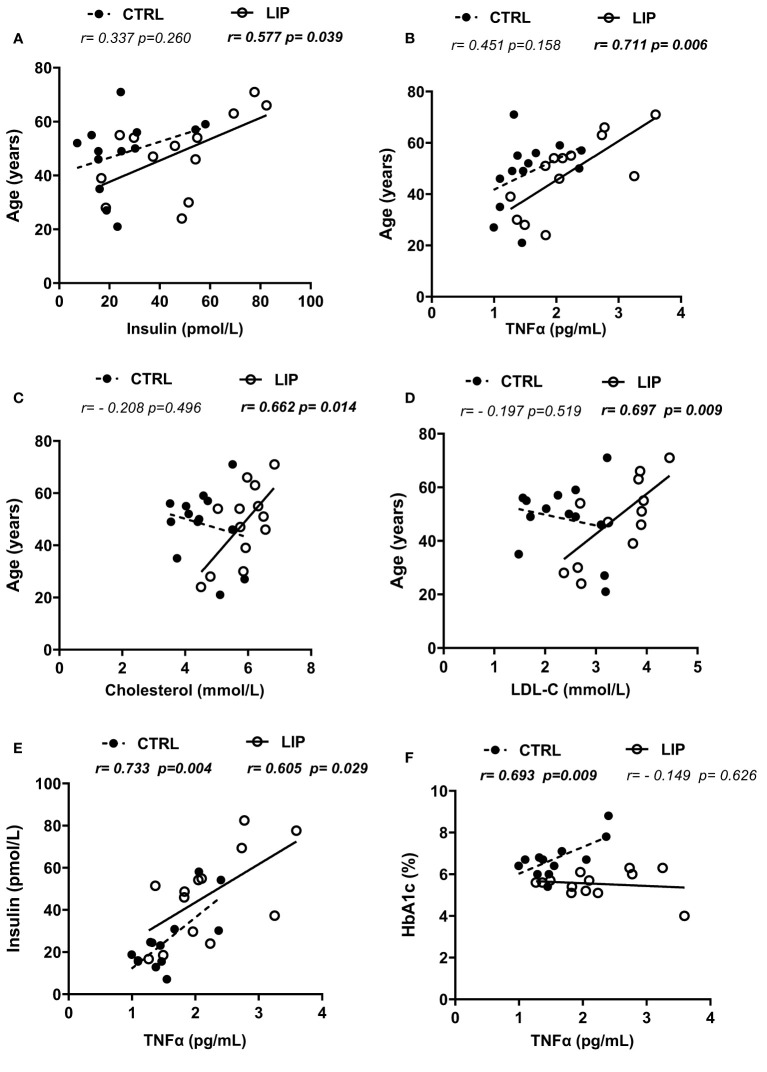
Correlations between biochemical markers and metabolic parameters in lipedema and control groups. **(A–D)**: association between age and circulating concentrations of insulin, TNFα, total cholesterol and LDL-C respectively; **(E)**: correlation between plasma insulin and TNFα levels; **(F)**: correlation between HbA1c percentage and TNFα plasma levels. r coefficients and p values are from Pearson’s pairwise correlations; HbA1c, Hemoglobin A1c; LDL-C, Low-density lipoprotein cholesterol; TNFα, Tumour Necrosis Factor-alpha; CTRL, Control group; LIP, Lipedema group.

The differences in metabolic status in lipedema compared to individuals with overweight and obesity were further investigated by performing correlation analyses between the distinct biomarkers and selected phenotypic parameters. We tested the hypothesis that associations between age and circulating parameters or among biomarkers are distinct for people with and without lipedema. Interestingly in the lipedema group, insulin (r=0.577; p=0.039), TNFα (r=0.741; p=0.004), total cholesterol (r=0.662; p=0.014) and LDL-C concentrations (r=0.693; p= 0.009) were positively associated with age while none of these correlations was significant for the control group (**Figures 1A–D**). In both groups, TNFα levels positively correlated with insulin concentrations (lipedema: r=0.605; p=0.029; control: r=0.733; p=0.004). Finally, TNFα concentration was positively associated with HbA1c in the control group (r=0.693; p=0.009) and not in the lipedema group (r=-0.149; p=0.626) (**Figures 1E, F**).

### Parameters of systemic inflammation in patients with lipedema

**Figure 2:**
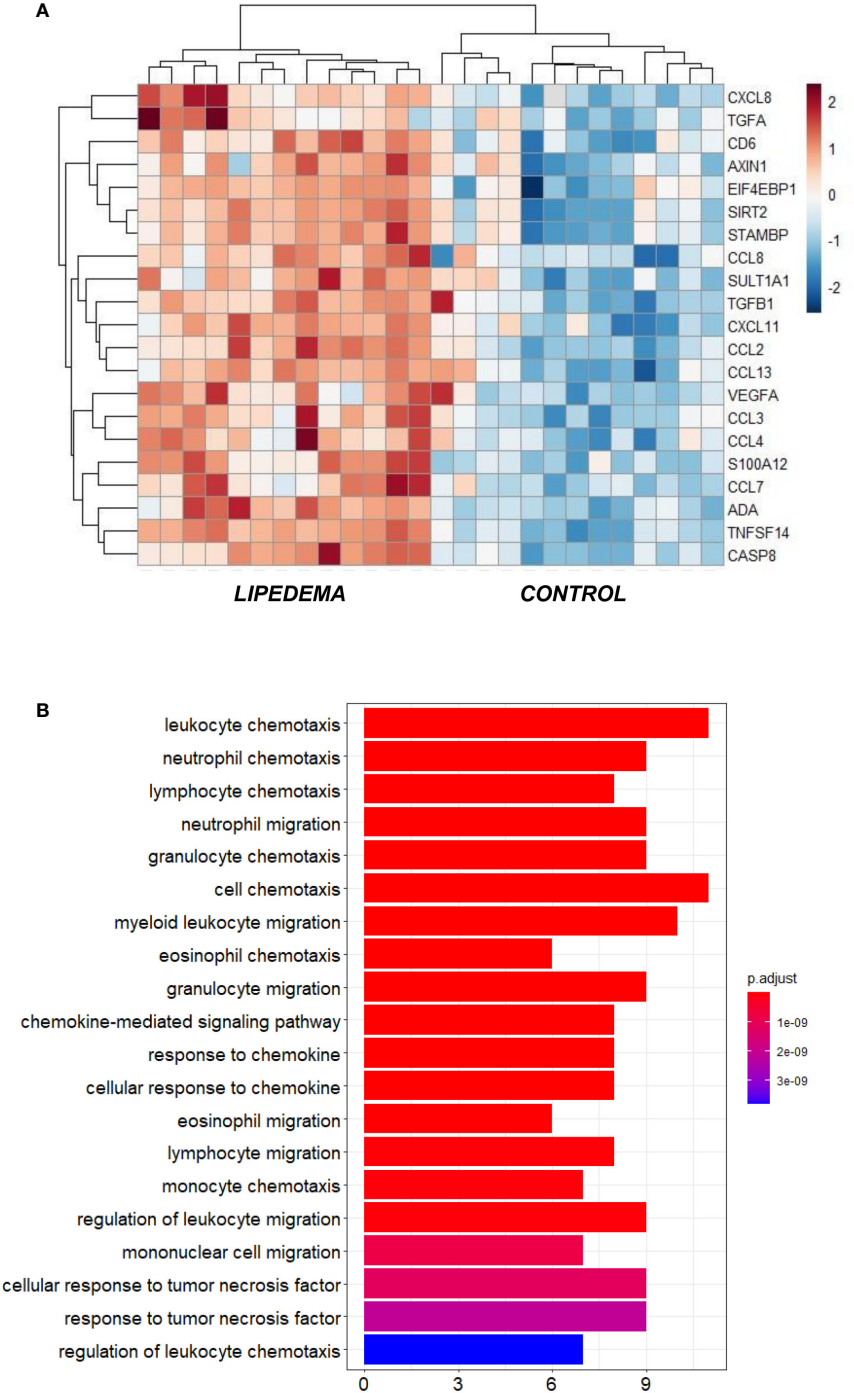
**(A)** Heatmap visualization of the significantly different inflammatory protein expression in plasma samples of lipedema compared to age- and BMI-matched control group (p_adj < 0.05); **(B)** Gene ontology enrichment analysis presenting the top 20 biological processes (GO terms) enriched by the differential expressed plasma inflammatory proteins between lipedema and control groups.

Given the higher levels of TNFα and oxidative stress markers in lipedema patients compared to the control group, we further sought to investigate the pattern of pro-inflammatory markers in both groups. Using a high-sensitivity protein expression multiplex proteomics method, we evaluated 92 proteins that play a role in the systemic inflammatory response. Out of the 92 plasma proteins, 14 were excluded from further analysis (IL-2RB, IL-1 alpha, IL-2, TSLP, IL-22 RA1, Beta-NGF, IL-24, IL-13, IL-20, IL-33, IL-4, LIF, NRTN, IL-5) due to the expression levels below the limit of detection in more than 30% of the samples for the given protein. Some of these proteins such as IL-1α, IL-2, TSLP, IL-22 RA1, IL-13, IL-20 and IL-33 have also been reported to be undetectable in both plasma and serum of lean and obese men and women (48). In our study, a total of 78 inflammatory proteins were further included in the analyses (85% detectability percentage). Interestingly, 21 inflammatory proteins were significantly upregulated in the plasma of lipedema patients compared to the control group (adjusted p <0.05), and none were higher in the control compared to the lipedema group (**Figure 2A**). The upregulated inflammatory proteins in lipedema sorted by lowest adjusted p-value consisted of TNFSF14, CASP8, EN-RAGE (S100-A12), EIF4EBP1, ADA, MCP-1, SIRT2, CXCL11, CCL3, STAMBP, CD6, MCP-3, CCL4, MCP-2, ST1A1, IL-8, LAP TGF-beta-1, AXIN1, MCP-4, TGF-alpha, VEGFA (**Figure 2A**; **Table 3**). We further investigated the biological processes and molecular activities represented by these differentially expressed circulating inflammatory proteins. Interestingly, the pathways enriched by these inflammatory proteins were consistently associated with immune cell chemotaxis and migration processes (adjusted p<0.01; **Figure 2B**). Therefore, a higher systemic inflammatory profile in lipedema could reflect the inflammatory state in the affected SAT, by an increased immune cell attraction and infiltration in this tissue.

**Table 3:** Detectable inflammatory markers measured by proteomic approach and significance level.

Gene Symbol	P-value	Adjusted p-value	Gene description
**TNFSF14**	1.9E-07	1.5E-05	Tumor necrosis factor ligand superfamily member 14 (TNFSF14)
**CASP-8**	1.9E-07	1.5E-05	Caspase-8 (CASP-8)
**EN-RAGE**	1.9E-07	1.5E-05	Protein S100-A12 (EN-RAGE)
**4E-BP1**	3.9E-07	2.8E-05	Eukaryotic translation initiation factor 4E-binding protein 1 (4E-BP1)
**ADA**	3.9E-07	2.8E-05	Adenosine Deaminase (ADA)
**MCP-1**	3.9E-07	2.8E-05	Monocyte chemotactic protein 1 (MCP-1)
**SIRT2**	7.7E-07	5.5E-05	SIR2-like protein 2 (SIRT2)
**CXCL11**	2.3E-06	0.0002	C-X-C motif chemokine 11 (CXCL11)
**CCL3**	3.7E-06	0.0003	C-C motif chemokine 3 (CCL3)
**STAMBP**	3.7E-06	0.0003	STAM-binding protein (STAMBP)
**CD6**	8.7E-06	0.0006	T cell surface glycoprotein CD6 isoform (CD6)
**MCP-3**	8.7E-06	0.0006	Monocyte chemotactic protein 3 (MCP-3)
**CCL4**	1.3E-05	0.0009	C-C motif chemokine 4 (CCL4)
**MCP-2**	1.9E-05	0.0012	Monocyte chemotactic protein 2 (MCP-2)
**ST1A1**	3.8E-05	0.0024	Sulfotransferase 1A1 (ST1A1)
**IL8**	5.2E-05	0.0033	Interleukin-8 (IL-8)
**LAP TGF-beta-1**	7.2E-05	0.0044	Latency-associated peptide transforming growth factor (LAP TGF-beta-1)
**AXIN1**	0.0001	0.0080	Axin-1 (AXIN1)
**MCP-4**	0.0002	0.0100	Monocyte chemotactic protein 4 (MCP-4)
**TGF-alpha**	0.0002	0.0100	Transforming growth factor alpha (TGF-alpha)
**VEGFA**	0.0002	0.0100	Vascular endothelial growth factor A (VEGF-A)
CD40	0.0012	0.0712	CD40L receptor (CD40)
CXCL6	0.0015	0.0868	C-X-C motif chemokine 6 (CXCL6)
CXCL1	0.0019	0.1053	C-X-C motif chemokine 1 (CXCL1)
OSM	0.0023	0.1269	Oncostatin-M (OSM)
CD5	0.0029	0.1492	T-cell surface glycoprotein CD5 (CD5)
MMP-1	0.0029	0.1492	Matrix metalloproteinase-1 (MMP-1)
IL-17C	0.0051	0.2536	Interleukin-17C (IL-17C)
NT-3	0.0051	0.2536	Neurotrophin-3 (NT-3)
CXCL10	0.0061	0.2977	C-X-C motif chemokine 10 (CXCL10)
CCL25	0.0086	0.4129	C-C motif chemokine 25 (CCL25)
LIF-R	0.0120	0.5628	Leukemia inhibitory factor receptor (LIF-R)
HGF	0.0164	0.7548	Hepatocyte growth factor (HGF)
CCL11	0.0191	0.8403	Eotaxin (CCL11)
CXCL5	0.0191	0.8403	C-X-C motif chemokine 5 (CXCL5)
IL-15RA	0.0221	0.9524	Interleukin-15 receptor subunit alpha (IL-15RA)
ARTN	0.0256	0.9598	Artemin (ARTN)
CCL19	0.0256	0.9598	C-C motif chemokine 19 (CCL19)
CCL20	0.0295	0.9598	C-C motif chemokine 20 (CCL20)
CCL23	0.0441	0.9598	C-C motif chemokine 23 (CCL23)
CCL28	0.0501	0.9598	C-C motif chemokine 28 (CCL28)
CD244	0.0501	0.9598	Natural killer cell receptor 2B4 (CD244)
CD8A	0.0642	0.9598	T-cell surface glycoprotein CD8 alpha chain (CD8A)
CDCP1	0.0908	0.9598	CUB domain-containing protein 1 (CDCP1)
CSF-1	0.0908	0.9598	Macrophage colony-stimulating factor 1 (CSF-1)
CST5	0.1129	0.9598	Cystatin D (CST5)
CX3CL1	0.1225	0.9598	Fractalkine (CX3CL1)
CXCL9	0.1254	0.9598	C-X-C motif chemokine 9 (CXCL9)
DNER	0.1254	0.9598	Delta and Notch-like epidermal growth factor-related (DNER)
FGF-19	0.1389	0.9598	Fibroblast growth factor 19 (FGF-19)
FGF-21	0.1534	0.9598	Fibroblast growth factor 21 (FGF-21)
FGF-23	0.1690	0.9598	Fibroblast growth factor 23 (FGF-23)
FGF-5	0.2035	0.9598	Fibroblast growth factor 5 (FGF-5)
Flt3L	0.2035	0.9598	Fms-related tyrosine kinase 3 ligand (Flt3L)
GDNF	0.2226	0.9598	Glial cell line-derived neurotrophic factor (GDNF)
IFN-gamma	0.2428	0.9598	Interferon gamma (IFN-gamma)
IL10	0.2642	0.9598	Interleukin-10 (IL10)
IL-10RA	0.2642	0.9598	Interleukin-10 receptor subunit alpha (IL-10RA)
IL-10RB	0.2869	0.9598	Interleukin-10 receptor subunit beta (IL-10RB)
IL-12B	0.3107	0.9598	Interleukin-12 subunit beta (IL-12B)
IL-17A	0.3107	0.9598	Interleukin-17A (IL-17A)
IL18	0.3358	0.9598	Interleukin-18 (IL-18)
IL-18R1	0.3358	0.9598	Interleukin-18 receptor 1 (IL-18R1)
IL-20RA	0.3358	0.9598	Interleukin-20 receptor subunit alpha (IL-20RA)
IL6	0.4184	0.9598	Interleukin-6 (IL6)
IL7	0.4184	0.9598	Interleukin-7 (IL-7)
MMP-10	0.5033	0.9598	Matrix metalloproteinase-10 (MMP-10)
OPG	0.5788	0.9598	Osteoprotegerin (OPG)
PD-L1	0.6498	0.9598	Programmed cell death 1 ligand 1 (PD-L1)
SCF	0.7241	0.9598	Stem cell factor (SCF)
SLAMF1	0.7623	0.9598	Signaling lymphocytic activation molecule (SLAMF1)
TNF	0.7623	0.9598	Tumor necrosis factor (TNF)
TNFB	0.8403	0.9598	TNF-beta (TNFB)
TNFRSF9	0.8403	0.9598	Tumor necrosis factor receptor superfamily member (TNFRSF9)
TRAIL	0.8798	0.9598	TNF-related apoptosis-inducing ligand (TRAIL)
TRANCE	0.9197	0.9598	TNF-related activation-induced cytokine (TRANCE)
TWEAK	0.9197	0.9598	Tumor necrosis factor (Ligand) superfamily, member 12 (TWEAK)
uPA	0.9598	0.9598	Urokinase-type plasminogen activator (uPA)

## Discussion

Using lipedema as a human model to understand mechanisms linking AT (impaired) expandability to metabolic outcomes, and given the current limited data availability on circulating biomarkers of lipedema, we aimed to systematically characterize the plasma profile of carefully selected lipedema patients and to identify the most differentially regulated molecules between lipedema and overweight or obesity patients. We found an unexpected dissociation of lower HbA1c and higher adiponectin in lipedema patients, despite higher fasting insulin concentrations and higher circulating parameters of liver function, inflammation and oxidative stress, compared to age- and BMI-matched women without lipedema. These data generate several novel hypotheses. Higher inflammation and oxidative stress parameters may reflect inflammatory processes in SAT. Activation of inflammatory pathways may subsequently contribute to peripheral insulin resistance that is compensated for by higher insulin secretion. Higher insulin secretion may contribute to lower glucose metabolism parameters in a proposed compensated state. Our data may alternatively suggest that the expected association between activation of inflammatory and oxidative stress pathways and impaired glucose metabolism are counterbalanced by protective factors such as higher adiponectin secretion. Although further studies are warranted to validate these proposed models, we find indications for both explanations, in our comparison between lipedema and control patients.

Lower rates of T2D have been reported in lipedema despite the excessive and disproportionate fat accumulation in this condition (6). We did not assess insulin sensitivity by euglycemic-hyperinsulinemic clamps or HOMA-IR and acknowledge that as one limitation of our study. Higher fasting plasma insulin in our cohort of lipedema patients suggests impaired insulin sensitivity, but in contrast, higher adiponectin plasma concentrations have been associated with improved peripheral and whole-body insulin sensitivity (49–52). Adiponectin is an adipokine almost exclusively secreted by adipocytes, and to a lesser extent during AT expansion in central obesity (53), and higher concentrations of adiponectin correlated with higher AT accumulation in lower extremities (54). Indeed, the influence of expanded AT on whole-body metabolic status is more related to the tissue location (AT distribution) than its total amount (AT mass) (54, 55). Thus, a gynoid distribution of AT has been suggested to be protective against the development of fat-related metabolic complications among which, insulin resistance (54–56). The lower level of HbA1C and reported reduced diabetes risk in lipedema could therefore result from the predominant fat accumulation in the lower body, in contrast to central fat accumulation in obesity.

In lipedema patients, we found higher plasma concentrations of total cholesterol and LDL-C compared to controls. This is similar to the findings of Felmerer et al. where serum levels of total cholesterol and LDL-C ranged from upper normal to pathological values in lipedema compared to BMI-matched controls (40). A less favorable lipid profile could be reflective, among others, of a dysregulated liver function or higher liver fat accumulation. Accordingly, ALAT concentrations were higher in lipedema patients, ranging from the upper half to the normal range. From these findings, we could not suggest a defect in liver function but rather indicative of deterioration in lipids metabolism or liver fat accumulation (57). Noteworthy, our phenotyping did not include analyses of liver fat content, which therefore requires further studies on ectopic fat accumulation despite lipedema. Although higher plasma levels of total cholesterol and LDL-C did not correlate with liver enzymes, these were positively associated with age in lipedema patients. Similarly, the plasma concentration of TNFα (established marker of systemic inflammation) positively correlated with age only in lipedema patients. Interestingly, there was a trend for increased lipedema stages with patients’ age. This suggests that the dysregulation in lipid metabolism and inflammatory state in lipedema may depend on the disease stage. The low number of patients in the different lipedema stages in this study prevents us to subdivide the data to verify this hypothesis.

Lipedema is a suggested inflammatory disease, mostly due to a chronic low-grade pro-inflammatory state in AT. Indeed, an increased immune cell infiltration (mostly macrophages) was shown in AT and skin samples of lipedema patients (36–38, 40, 41). A trend of increase in inflammatory gene expression was also found in adipocytes differentiated from lipedema adipose precursor cells (38). However, fewer studies have investigated systemic inflammation in lipedema. Slightly elevated concentrations of three (IL-11, IL-28A and IL-29) out of 39 evaluated inflammatory proteins were recently shown in the serum of lipedema patients (58). In contrast, no changes in plasma levels of IL-6 were found in lipedema compared to BMI-matched controls (40). We found that 21 of the 78 circulating inflammatory proteins evaluated in our cohort were highly expressed in lipedema compared to obese controls supporting a strong systemic pro-inflammatory signature in lipedema compared to the well-described low-grade inflammation characterizing obesity. Notably, the pathways enriched by these inflammatory proteins consisted of immune cell chemotaxis and migration, suggesting that increased pro-inflammation in lipedema patients could derive from the previously shown increased immune cell infiltration in lipedema SAT (37, 40, 41).

Elevated levels of systemic inflammatory cytokines may not only reflect an increased immune cell infiltration in lipedema SAT but also an impairment in processes related to the vascular system (37). For instance, the cytokines VEGFA, TGFα and TGFβ1 were among the inflammatory markers upregulated in lipedema. VEGF is a lymphatic-related cytokine and marker of angiogenesis, which has been related to increased macrophage infiltrate, inflammation, alteration of vascular permeability and lipedema progression (41, 58, 59). In addition, TGFα is involved in angiogenesis-related processes and inflammation (60, 61), while TGFβ is involved in fibroblast growth, collagen production, fibrosis and angiogenesis (62, 63). In response to excess lipids accumulation, AT mass expansion necessitates extensive remodelling of the extracellular matrix and angiogenesis which in turn require inflammation in the adipocyte microenvironment (64, 65). Therefore the higher inflammatory state described in lipedema AT by other studies could be the consequence of the tissue expansion as observed in lipedema rather than a pathological state in the tissue *per se*. This could also explain the lack of association between higher inflammation and insulin resistance in lipedema, but the mechanisms that may protect lipedema patients from developing diabetes warrant further investigations.

Increased inflammation can also influence oxidative stress status (31, 47). Accordingly, we show increased oxidative stress in lipedema, determined by elevated concentrations of MDA a surrogate measure of lipids peroxidation in the circulation. One previous study described increased oxidative stress in lipedema with higher serum concentrations of MDA and plasma protein carbonyls in lipedema (59). High levels of MDA may indicate severe preexisting oxidative stress which could be found in long-standing adipocyte inflammatory processes and likely represent an accelerated lipid peroxidation in lipedema AT (66). In addition to high MDA levels, we show an upregulation of antioxidant enzymes catalase and SOD. Oxidative stress results from an imbalance between the production of reactive oxygen species (ROS) or pro-oxidant, and antioxidant enzymes to clear the ROS and maintain the oxidative stress balance (67). Higher levels of antioxidant enzyme activity could be a compensatory response to higher ROS production (67, 68). Oxidative stress can cause damage in AT which has been associated with the activation of stress signalling pathways, impaired adipogenesis, autophagy and apoptosis (31). This could lead to dysregulated adipocytokine signalling, and further increase immune cell infiltration and inflammation (31). In turn, inflammatory cytokines can increase oxidative stress in AT (47). This would generate a vicious cycle between oxidative stress and inflammation, further aggravating the pro-inflammatory state in lipedema. Therefore, oxidative stress might play a pivotal role in the development, or the clinical manifestations of lipedema.

Although this study established differences in circulating markers between lipedema and overweight and obesity, it was limited by the small number of patients and the lack of uniformity in the patient’s menstrual cycle during blood sample collection, particularly for the evaluation of circulating sex hormones and the lack of more insulin sensitivity/resistance measures. Moreover, the pre- vs post-menopausal status of the patients was not considered in this study. These limitations were compensated by the comparable phenotypes of the patients involved (gender, age and BMI) and the extensive measurements of the several circulating biomarkers in both groups, making our findings novel and contributing to advancing the understanding of lipedema pathophysiology.

## Conclusion

Patients affected with lipedema have a seemingly preserved glycemic control when compared to obesity, but higher concentrations of total and LDL-cholesterol, ALAT, and inflammatory and oxidative stress parameters. We did not find a single parameter with clinical relevance for lipedema diagnosis. However, we suggest several inflammatory and oxidative stress biomarkers which might reflect the disease, as they are differentially regulated in lipedema compared to overweight and obesity. Further studies are needed to understand whether the most different inflammatory markers between these groups (TNFSF14, CASP8, EN-RAGE, EIF4EBP1, ADA, MCP-1) play a mechanistic role in lipedema development and perpetuation. Although higher circulating inflammation may reflect extensive changes in AT processes, the development and clinical manifestation of lipedema may not be limited to AT function given the other associated symptoms like pain. Therefore, further research studies with a larger sample size should be performed and include AT biopsies and possibly other tissue or systems such as vascular and central nervous systems.

## Data availability statement

The raw data supporting the conclusions of this article will be made available by the authors, without undue reservation.

## Ethics statement

The studies involving human participants were reviewed and approved by Ethics Committee of the University of Leipzig. The patients/participants provided their written informed consent to participate in this study.

## Author contributions

MB and MC conceived the study. PN, MC and NK generated the data. PN analyzed the data. PN and MB wrote the first draft of the manuscript. All authors contributed to the article and approved the submitted version.

## Acknowledgments

We thank all women that participated in this study. We also thank the nurses, the clinical and laboratory staff of Ly.search GmbH and the Medical Department III of the University Hospital of Leipzig who assisted with the sample collection and analyses.

## Conflict of interest

MB received honoraria as a consultant and speaker from Amgen, AstraZeneca, Bayer, Boehringer-Ingelheim, Lilly, Novo Nordisk, Novartis and Sanofi. MC is the research director of Ly.Search GmbH, Germany.

The remaining authors declare that the research was conducted in the absence of any commercial or financial relationships that could be construed as a potential conflict of interest.

## Publisher’s note

All claims expressed in this article are solely those of the authors and do not necessarily represent those of their affiliated organizations, or those of the publisher, the editors and the reviewers. Any product that may be evaluated in this article, or claim that may be made by its manufacturer, is not guaranteed or endorsed by the publisher.
